# Serum Vitamin D as a Marker of Impaired Information Processing Speed and Early Disability in Multiple Sclerosis Patients

**DOI:** 10.3390/brainsci11111521

**Published:** 2021-11-17

**Authors:** Eleonora Virgilio, Domizia Vecchio, Ilaria Crespi, Paolo Barbero, Beatrice Caloni, Paola Naldi, Roberto Cantello, Umberto Dianzani, Cristoforo Comi

**Affiliations:** 1 Neurology Unit, Department of Translational Medicine, Maggiore Della Carità Hospital, University of Piemonte Orientale, 28100 Novara, Italy; domizia.vecchio@gmail.com (D.V.); paolo91b@gmail.com (P.B.); 20009588@studenti.uniupo.it (B.C.); pnaldi@inwind.it (P.N.); roberto.cantello@med.uniupo.it (R.C.); 2 Ph.D. Program in Medical Sciences and Biotechnologies, Department of Translational Medicine, University of Piemonte Orientale, 28100 Novara, Italy; 3 Neurology Unit, Department of Translational Medicine, S. Andrea Hospital, University of Piemonte Orientale, 13100 Vercelli, Italy; cristoforo.comi@med.uniupo.it; 4 Interdisciplinary Research Center of Autoimmune Diseases (IRCAD), Department of Health Sciences, University of Piemonte Orientale, 28100 Novara, Italy; umberto.dianzani@med.uniupo.it; 5 Clinical Biochemistry, Department of Health Sciences, University of Piemonte Orientale, 28100 Novara, Italy; ilaria.crespi@maggioreosp.novara.it

**Keywords:** multiple sclerosis, vitamin D, cognition, information processing speed, IPS, symbol digit modalities test, SDMT

## Abstract

Slowed information processing speed (IPS) is the hallmark and first cognitive domain to be altered in multiple sclerosis (MS) patients. Insufficient serum vitamin D was previously associated with disease development, relapses, and progression, but little is reported on cognition. However, vitamin D and cognitive impairment (CI) in other neurodegenerative diseases have already been linked. We explored the possible correlation between vitamin D and IPS at diagnosis and early disability at last follow-up in 81 MS patients. At diagnosis, we collected vitamin D levels and performed a Symbol Digit Modalities Test (SDMT). Raw scores were adjusted for age, gender, and educational level. Early disability was evaluated with MS severity score (MSSS) and age-related MSSS (ARMSS). A total of 71 patients (86.58%) showed hypovitaminosis D (19.71 ± 8.76 ng/mL) and 18 patients (21.95%) had CI. Patients with CI showed severe hypovitaminosis D (*p* = 0.004). No patients with sufficient vitamin D levels had CI. We found a positive correlation between vitamin D levels at diagnosis and (1) SDMT raw and *z*-score that persisted after correction for sunlight exposure and MRI baseline characteristics, and (2) EDSS, MSSS, and ARMSS after a mean 2 year follow-up. Low vitamin D levels may affect both cognition and early disability in newly diagnosed MS patients.

## 1. Introduction

Multiple sclerosis (MS) is a chronic inflammatory disease of the central nervous system (CNS). Clinical manifestations range from sensory and motor symptoms to blurred vision, brainstem syndrome, and cognitive impairment (CI) [1,2,3]. In the past decades, CI was underestimated in MS patients and thought to appear only in primary progressive (PP) and secondary progressive (SP) disease stages [1]. Today, it is well established that CI affects a large proportion of MS patients from onset to all disease stages [1,2,3]. Slowed information processing speed (IPS) is the hallmark of CI in MS and the first cognitive domain to be altered at the diagnosis, but CI in MS patients may also display impaired verbal and visuospatial memory [1,2,3]. IPS can be evaluated using different tests such as the Paced Auditory Serial Addition Task (PASAT) and Symbol Digit Modalities Test (SDMT) [1,4]. Currently, SDMT is the recommended test for IPS evaluation in newly diagnosed MS patients [1,2]. SMDT is included in several neuropsychological test batteries such as the Brief International Cognitive Assessment in MS patients (BICAMS) test battery, which is an internationally validated test battery easy and quick to administrate. BICAMS includes SDMT and California Verbal Learning Test-II (CVLT-II) as a measure of verbal memory and Brief Visuospatial Memory Test-Revised (BVMT-R) to evaluate visuospatial memory [2]. Even though neurologists have become more aware of CI from MS diagnosis, the mechanisms underlying CI in MS are not yet well recognized. In particular, IPS is probably not the consequence of a specific brain lesion but is more likely the result of a network disconnection syndrome [5,6]. Imaging studies have provided evidence of the role of white-matter demyelination, as well as focal inflammation, in cognition [7,8,9,10]. However, the extent of white-matter abnormalities did not fully explain CI in MS patients [10,11]. Gray-matter pathology and focal/global gray-matter volume correlated with cognition in several studies [9,10,11], but key processes for neurodegeneration development are still unknown. Although, in the last decade, several disease-modifying treatments (DMTs) have been introduced in clinical practice, their efficacy on cognition has still not been proven [12].

Vitamin D is a molecule involved in several cellular processes from bone density to immune system regulation [13,14]. The effects of vitamin D on resident CNS neuronal and immune cells are especially relevant in promoting neuronal survival mediated by reduced proinflammatory cytokine and increased neuronal growth factors [13,15,16]. In B and T lymphocytes, as well as oligodendrocytes, neurons, and microglia, vitamin D binds a specific receptor, creating a complex that modulates gene transcription in target cells [15,16]. Evidence linked insufficient serum vitamin D and vitamin D genetic polymorphisms with a higher risk of MS, and sunlight exposure proved a protective effect, likely secondary to the immunomodulatory effects of the vitamin [16,17,18,19,20]. Additionally, patients with vitamin D deficiency experience greater disease activity both clinically and radiologically, as well as greater progression in terms of EDSS, which was not confirmed in a few studies [18,21,22,23,24,25]. Lack of vitamin D was also linked with a higher conversion rate from clinically isolated syndrome (CIS) to clinically defined MS [25,26]. By contrast, several clinical trials failed to prove vitamin D as an effective add-on DMT [24], even though an increase in TGF-beta levels was noted among patients under supplementation, supporting the immunomodulatory effects on MS prognosis by promoting T-reg differentiation [25]. Few studies explored the possible correlation between CI and vitamin D in the MS population [27,28,29], and trials on vitamin D supplementation so far have not included cognitive evaluation [23,30]. 

Furthermore, the association between serum vitamin D levels and CI has already been established in other neurodegenerative diseases such as cortical (i.e., Alzheimer’s disease) and subcortical dementia (i.e., Parkinson’s disease) [31]. In animal models, vitamin D inhibits beta-amyloid accumulation, promotes clearance of the beta-amyloid peptide, and reduces neuronal death in the hippocampus [32,33]. Moreover, a correlation has been shown between vitamin D levels and CI in other autoimmune diseases, such as systemic lupus erythematosus (SLE) [32].

The primary aim of this study was to explore the possible correlation between vitamin D and cognition, particularly IPS, early in the disease, precisely at MS diagnosis, using the SDMT. The secondary aim was to confirm the relationship between vitamin D and early disability at the last clinical follow-up at least 1 year after MS diagnosis. 

## 2. Materials and Methods

### 2.1. Study Design and Population

We retrospectively collected data from 81 newly diagnosed MS patients from our MS Center in Novara. We included patients with MS diagnosis according to 2010 or 2017 McDonald criteria [34,35]. We selected patients displaying both serum vitamin D sampling and SDMT assessment at diagnosis, at least after 1 year of follow-up. We excluded patients with psychiatric, gastrointestinal, and other neurological comorbidities, treated with steroids at the time of cognitive evaluation or sampling time, with disorders related to vitamin D deficiency such as parathyroid diseases, during pregnancy, or breastfeeding. Dietary information, smoking, and body mass index (BMI) at sampling time were available for only a small subgroup of patients; therefore, those data were not included in the analysis. Finally, to ensure homogeneity in terms of sunlight exposure, patients were recruited from two northwestern Italian regions with similar climates, categorized into spring/summer versus autumn/winter sampling. All patients were Caucasians. We collected at diagnosis clinical data (sex, age of onset, age at diagnosis, MS course, and EDSS) and imaging data (brain and spinal cord MRI). Baseline MRI scans were performed within 3 months of lumbar puncture (LP) according to Italian diagnostic work-up recommendation for clinical practice [36]. We considered the T2 white-matter lesion load (WMLL), using an arbitrary cutoff of 10 lesions to define high and low WMLL, presence or absence of spinal cord lesions (SL), and presence or absence of gadolinium-enhancing (Gd+) lesions. Exposure to DMTs during follow-up was also recorded. Early disability at last clinical follow-up was evaluated with expanded disability status score (EDSS), MS severity score (MSSS) [37], and age-related MSSS (ARMSS) [38]. An informed consent form for both diagnostic and research purposes was signed by all patients at the time of the LP.

### 2.2. Vitamin D and Cognitive Evaluation

Vitamin D was usually assessed on the same day patient underwent LP. Vitamin D was obtained with chemiluminescence (CLIA) in the same biochemistry department and measured in nanograms per milliliter (ng/mL). We used the LIAISON^®^ 25OH Vitamin D total assay certified since 2014 (DiaSorin Inc., 1951 Northwestern Ave—Stillwater, MN 55082—USA). The assay is fully automated. The kit has a range of detection between 4.0 and 150 ng/mL. Samples were analyzed by board-certified laboratory technicians, blinded to clinical data. All experimental measurements were performed according to manufacturers’ instructions. We defined three categories for vitamin D status according to the Italian Endocrinologist Guidelines [39]: (1) vitamin D deficiency when the concentration resulted below or equal to 20 ng/mL (corresponding to 50 nmol/L), (2) vitamin D insufficiency when the concentration was between 20 and 30 ng/mL, and (3) vitamin D normality when the levels were above 30 ng/mL (corresponding to 75 nmol/L).

We performed statistical analysis considering firstly vitamin D in the three categories and subsequently joining deficient and insufficient vitamin D in one group opposed to normal levels. IPS was evaluated with SDMT in the oral form as suggested by clinical practice guidelines [1]. SMDT was performed within 3 months of LP execution. Raw values were corrected for age, gender, and educational levels using the Italian normative data for BICAMS [2], and normalized *z*-scores were obtained.

### 2.3. Statistical Analysis

Statistical analysis was performed using SPSS 25.0 for Windows (SPSS Inc., Chicago, IL, USA) and Graphpad Prism 9 for Windows (Graphpad Software, La Jolla, CA, USA). We presented continuous data as mean and standard deviation (SD), categorical data as median, range, and interquartile range (IQR), and proportions as numbers with the corresponding percentage. The normal distribution of data was preliminarily assessed with the Kolmogorov–Smirnov test. Unpaired *t*-test with Welch’s test, Mann–Whitney U test, and Kruskal–Wallis test were used for comparison between continuous variables; chi-squared test and Fisher test were used for categorical variables. Spearman’s rank correlation coefficient test was used for the correlation between continuous variables. Multiple regression analyses including vitamin D level, age, gender, educational level, EDSS, type of MS, and MRI characteristics at baseline as independent variables; SDMT raw value and *z*-score were chosen as dependent variables to identify the best predictors of slowed IPS. In all analyses, we considered *p* < 0.05 as statistically significant. 

## 3. Results

Of our 81 enrolled patients, 54 (67%) were female and 27 (33%) were male. Of them, 71 (88%) were relapsing–remitting (RR) MS patients, two (2%) had clinically isolated syndrome (CIS), seven (9%) had radiologically isolated syndrome (RIS), and one (1%) was a primary progressive (PP) patient. We enrolled patients with a mean age at diagnosis of 37.6 years (SD ± 11.7).

Most patients displayed low vitamin D (mean concentration of 19.71 ng/mL (SD ± 8.76)); only 10 patients (12.3%) had values ≥ 30 ng/mL, 46 (56.8%) had deficiency, and 25 (30.9%) had insufficiency. Forty-eight patients (59%) were sampled in winter, and 33 (41%) were sampled in summer; no differences were observed in mean levels of vitamin D between the two groups (19.3 ng/mL versus 20 ng/mL; *p*-value > 0.05).

**Figure 1:**
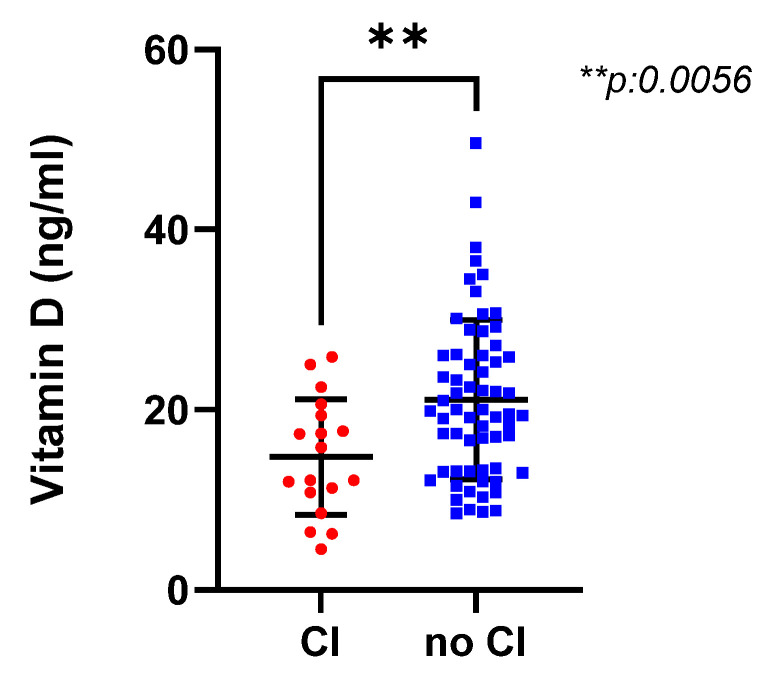
Scatter plots showing mean values and standard deviations of serum vitamin D at diagnosis in patients with preserved and impaired cognitive function (CI), defined as slowed information processing speed evaluated by the Symbol Digit Modalities Test. Patients with CI displayed significant lower values of vitamin D (*p =* 0.0056), and no patient with CI showed a normal value of vitamin D.

Eighteen patients (21.95%) had slowed IPS defined as with *z*-score < −1.5 (according to normative values) [2]. Of them, 15/18 (83.5%) were RRMS subtype, whereas 1/18 (5.5%) had RIS, 1/18 (5.5%) had CIS, and 1/18 (5.5%) was PP. In Table 1, we report a comparison of vitamin D levels based on demographic, clinical, and radiological characteristics. Patients with low vitamin D (i.e., deficiency + insufficiency) obtained significantly lower results at SMDT compared to patients with normal vitamin D No patients with CI showed normal vitamin D levels (represented in Figure 1).

**Figure 2:**
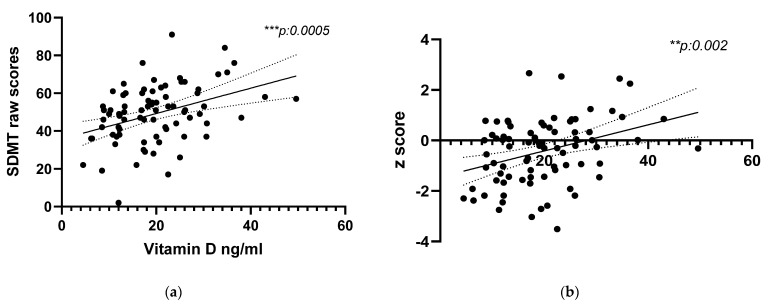
Correlation between serum vitamin D and SMDT raw values (**a**) and *z*-scores (**b**). SDMT is the neuropsychological test used to evaluate information processing speed. *z*-scores are normalized scores obtained after correction for age, sex, and educational levels according to the Italian normative values. We found a statistically significant positive correlation in both cases (*r*s: 0.38, *p*-value: 0.0005; *r*s: 0.33, *p*-value: 0.002).

We confirmed the relationship between hypovitaminosis D and slowed IPS by also performing a univariate analysis, as highlighted in Figure 2A for raw values (*r*s: 0.38, *p*-value: 0.0005) and B for *z*-scores (*r*s: 0.33, *p*-value: 0.002). This association was confirmed after correction for sunlight exposure (*r*: 0.392, *p*: 0001 raw values; *r*: 0.355, *p*: 001 with *z*-scores) and after correction for MRI parameters (*r*: 0.38, *p*: 0.01 raw values; *r*: 0.339, *p*: 0.002 with *z*-scores). 

**Figure 3:**
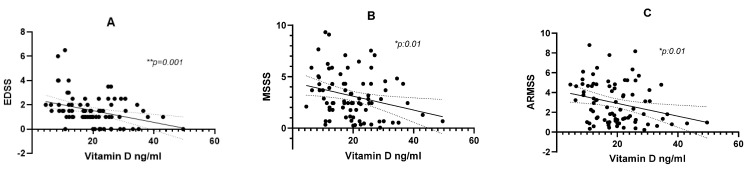
Correlation between serum vitamin D and EDSS (**A**), MSSS (**B**), and ARMSS (**C**). Vitamin D was statistically correlated with all three disability scores obtained at last clinical follow-up ((**A**) *r*s: −0.34, *p*: 0.001; (**B**) *r*s: −0.28, *p*: 0.01; (**C**) *r*s: −0.27, *p*: 0.01).

Moreover, low-vitamin-D patients were characterized by significantly higher EDSS at both baseline (*p*-value: 0.01 and 0.009) and the last clinical follow-up (*p*-value: 0.03) than the EDSS of patients with normal vitamin D. In fact, regarding the correlation with early disability, as shown in Figure 3, we confirmed that patients with low vitamin D developed higher EDSS (*r*s: −0.34, *p*: 0.001; Figure 3A), MSSS (*r*s: −0.28, *p*: 0.01; Figure 3B), and ARMSS (*r*s: −0.27, *p*: 0.01; Figure 3C). 

We explored the possible influence of MRI and disease activity on IPS results. Patients with Gd+ lesions or spinal cord involvement did not display more slowed IPS with raw values or corrected values (see Table 2), whereas patients with high brain WMLL had worse performance in SDMT.

Lastly, multiple regression analysis was statistically significant (adjusted *R*^2^: 0.30, *p*: 0.000), with educational levels (Beta: 0.346, 95% CI: 0.593–2.640, *p*: 0.002), vitamin D levels (Beta: 0.267, 95% CI: 0.109–0.820, *p*: 0.01), and age at diagnosis (Beta: −0.244, 95% CI: −0.574 to −0.056, *p*: 0.018) as the best predictors of raw scores of SDMT. Using SMDT *z*-scores as the independent variable and EDSS, type of MS, and MRI characteristics as dependent variables, multiple regression analysis was significant (adjusted *R*^2^: 0.114, *p*: 0.019) and vitamin D levels remained the only predictor of corrected IPS (Beta: 0.351, 95% CI: 0.019–0.085, *p*: 0.003). Multiple regression analyses are extensively reported in the Supplementary Materials. 

## 4. Discussion

Our study supports the hypothesis that vitamin D may influence cognition and disability from the early stages of MS disease. Hypovitaminosis D is frequently reported in the European population, especially in Italy, Spain, and some eastern European countries [40] with variable prevalence among studies on a healthy population (3% to 80%), due to age ranges, different lifestyles, skin pigmentation, dietary intake, season, latitude, and health status, which all affect vitamin D levels [41]. Hypovitaminosis D is more evident in elderly postmenopausal women [40,42], children, and pregnant women [41]. In MS, vitamin D is known to be a disease risk factor [17], whereas the association with relapse rate and disability is still under debate [18,21,22]. In our study, conducted in northwest Italy, only 12% of patients had values above 30 ng/mL and, therefore, did not need a supplementation. These data are in agreement with case–control studies conducted in Switzerland [43] and north Portugal [44], as well as with a retrospective study conducted on CIS in Lombardy [26] (52% CIS patients with deficiency versus 56.8% MS patients in our study). 

CI in the MS population is often underestimated and characterized by slowed IPS, as well as impaired visuospatial and verbal memory [1,2,3]. CI may influence the quality of life and treatment compliance, and it overall contributes to disability accumulation in MS patients [1,45]. Mechanisms underlying slowed IPS in MS are still uncertain and are probably linked to a cortical–subcortical disconnection as a result of focal gray- and white-matter demyelination, as well as neuronal and brain volume loss [5,6,7,9,11]. An impairment in IPS was described simultaneously with Gd+ lesions, suggesting that cognition may also be influenced by focal inflammation [8,46]. CI was reported to range from 20% to 65–70% as a function of different disease subtypes, disease duration, and NPS test battery [2,3,47]. Similar CI patterns were previously reported in CIS compared to RRMS [3] without significant differences, although the frequency of CI tends to be higher in RRMS than in CIS (possibly due to the younger age of CIS patients) [3]. However, most of the previous studies reported that IPS is one of the most frequent cognitive domains to be altered [1,2,3], and SMDT is the screening NPS test recommended at diagnosis in clinical practice [1]. In our study, we found only 21.9% of patients with impaired IPS at diagnosis, which is still comparable with published results. Nonetheless, we also included in our population seven patients with RIS and two patients with CIS, and we did not test for other cognitive domains (i.e., visuospatial memory, fluency, or verbal memory).

It is noteworthy that we observed that patients with low vitamin D obtained significantly lower results at SMDT compared to patients with normal vitamin D. These differences were noticeable in both raw scores and corrected *z*-scores, and no patients with CI showed normal vitamin D levels. We confirmed a correlation between hypovitaminosis D and slowed IPS independent of sunlight exposure or MRI baseline characteristics. This relationship was evident for both raw scores and *z*-scores.

Few data are available in the literature regarding vitamin D and cognition in MS, and cognition was not included in the so far inconclusive trials on vitamin D as an add-on treatment in MS. Recently, Darvish et al. showed an improvement in visuospatial memory and global cognition (evaluated by the Montreal Cognitive Assessment) scores after 3 months of vitamin D supplementation in 60 RRMS patients treated with interferon-beta; even if no differences were observed in SDMT results [28], these data support a beneficial effect of vitamin D on cognitive function in patients with both low and normal vitamin D at baseline [28]. Later, the same authors retrospectively explored IPS and MRI brain volume changes at two timepoints in 78 MS patients at baseline and 70 patients after a minimum follow-up of 9 months. Patients were recruited in Lebanon with a mean disease duration of 6 years. Patients with deficient vitamin D had lower SDMT scores which improved as a function of vitamin D. Brain volume analysis showed significant changes over time only in patients with sufficient vitamin D [27]. Similarly, they demonstrated slower IPS in patients with vitamin D deficiency [27]; however, the disease duration and demographic characteristics were very different since we included Caucasian patients at diagnosis before the introduction of DMTs. Alhussain et al. also explored cognition (using the Cambridge Neuropsychological Test Automated Battery to assess visuospatial and verbal memory, executive and attentive functions, decision making, and social cognition) in 39 Saudi MS patients in relation to vitamin D levels [29]. Vitamin D correlated with visuospatial memory, and EDSS was higher in patients with low vitamin D, but a specific test for IPS evaluation was not included [29]. In our population, we also found a correlation between vitamin D levels and early disability evaluated by EDSS, MSSS, and ARMSS. Lastly, Cortese et al., showed that, in 278 CIS patients, higher vitamin D levels predict better IPS results using PASAT in an 11 year follow-up of patients who participated in the BENEFIT trial [23]. In addition to IPS, a cognitive function that was previously reported to correlate with vitamin D levels in the MS population is visuospatial memory, which can be evaluated by BVMT-R included in BICAMS test battery. Unfortunately, only 65/81 of our patients presented a completed BICAMS battery; therefore, no results for verbal memory and visuospatial memory are included in our study.

Higher EDSS, increased age, and progressive MS were previously associated with the worst IPS performance [47,48,49], and higher education was associated with better SDMT performance [50]. Our results support these published data (except for differences with progressive MS not evaluable in our population). Moreover, other reports showed that low vitamin D levels correlated with greater disability, a more active disease course, and higher relapse rates, albeit with some conflicting results [18,21,22,23,24,25,26]. We support the hypothesis that hypovitaminosis may contribute to the development of higher disability evaluated with EDSS, MSSS, and ARMSS. EDSS is a disability score highly influenced by the motor performance. Nevertheless, we acknowledge that the degree of correlation is only weak–moderate, indicating that vitamin D is only partially responsible for motor disability accumulation. 

Lastly, Gd+ lesions and WMLL were previously associated with impaired cognition [7,8,46]. We confirmed that patients with high WMLL at diagnosis achieved worse cognitive performances, but no differences were observed when comparing patients in terms of focal activity. MRI LL probably influences cognition; however, in our cohort, MRI characteristics were not significant predictors in multiple regression analysis, in contrast to vitamin D. This is in line with previous studies where MRI WMLL was only partially responsible for CI in MS [7,10,11].

Our study had some limitations. Smoking, BMI, and dietary data that may influence vitamin D were not collected for all patients and, therefore, not included in the final analysis. Similarly, we did not exclude B12, folate deficiency, or thyroid dysfunctions that may have possibly affected cognition and IPS. Lastly, advanced MRI measures (such as brain volume) were not included in the multivariate model. Temporal and thalamic atrophy, as well as global brain atrophy, influences cognition and IPS; therefore, our results on vitamin D need to be replicated including those parameters [10,11].

## 5. Conclusions

In conclusion, our data support the hypothesis that vitamin D is involved in cognition in MS. This vitamin reduces CNS inflammation and promotes neuronal survival, suggesting that inadequate vitamin D levels could affect neuroaxonal integrity and the immune system, contributing to short-term effects on cellular homeostasis and long-term effects on neuronal loss. These processes in individuals with MS, consequently, may reflect slowed IPS upon diagnosis, which may influence their quality of life.

## Figures and Tables

**Table 1:** Between-group differences in vitamin D levels and demographic, clinical, and radiological characteristics (*N* = 81).

	Patients with Low Vitamin D		(3) Patients with Normal Vitamin D ≥30 ng/mL *N* = 10	*p*-Values (1) + (2) vs. (3)
	(1) Deficiency < 20 ng/mL *N* = 46	(2) Insufficiency ≥ 20 ng/mL *N* = 25		(1) vs. (2) vs. (3)
**Age at diagnosis** **Mean ± SD**	37.63 ± 12.07		37.50 ± 9.82	0.9
	39.56 ± 12.31	34.08 ± 10.99		0.2
**Age at onset** **Mean ± SD**	35.01 ± 11.84		36.80 ± 9.58	0.5
	37.61 ± 12.32	30.24 ± 9.37		***0.04***
**Gender F/M**	47/24		7/3	0.8
	31/15	16/9		0.9
**Edss at diagnosis** **Mean ± SD**	1.58 ± 0.85		0.85 ± 0.62	***0.01***
	1.67 ± 0.79	1.42 ± 0.94		***0.009***
**MRI high brain LL yes/no**	32/39		3/7	0.3
	20/26	12/13		0.6
**MRI spinal yes/no**	44/27		9/1	0.08
	29/17	15/10		0.2
**MRI Gd+ yes/no**	35/36		5/5	0.9
	23/23	12/13		0.9
**Vitamin D** **Mean ± SD**	17.29 ± 6.25		36.11 ± 6.16	***<0.0001***
	13.71 ± 4.15	24.15 ± 2.78		***<0.0001***
**SDMT raw score** **Mean ± SD**	47.58 ± 14.74		59.7 ± 15.16	***0.03***
	45.5 + 14.15	52.45 ±14.69		***0.02***
**Z-score** **Mean ± SD**	−0.55 ± 1.26		0.47 ± 1.28	***0.02***
	−0.67 ± 1.21	−0.26 ± 1.32		***0.03***
**EDSSS last fu** **Mean ± SD**	1.62 ± 1.26		0.95 ± 0.92	0.09
	1.84 ± 1.29	1.22 ± 1.12		***0.03***
**MSSS at last fu** **Mean ± SD**	3.23 ± 2.22		2.35 ± 1.82	0.3
	3.59 ± 2.15	2.58 ± 2.23		0.07
**ARMSS at last fu** **Mean ± SD**	3.06 ± 2.10		1.93 ± 1.55	0.1
	3.23 ± 2.07	2.74 ± 2.16		0.1

Abbreviations: ARMSS, age-related MS severity score; EDSS, Expanded Disability Status Scale; fu, follow-up; Gd+, gadolinium-enhanced lesions; LL, lesion load; MRI, magnetic resonance imaging; MS, multiple sclerosis; MSSS, MS severity score; SD, standard deviation; SDMT, Symbol Digit Modalities Test.

**Table 2:** Between-group differences in SDMT and radiological characteristics (*N* = 81).

	**SDMT Raw Scores** **Mean ± SD**	***p*-Value**
**Gd+ lesion (*N* = 40)** **Gd− lesion (*N* = 41)**	51.33 ± 14.41	0.29
	46.88 ± 15.85	
**High brain LL yes (*N* = 46)** **High brain LL no (*N* = 23)**	46.09 ± 16.59 53.0 ± 12.41	**0.02**
**MRI spinal yes (*N* = 53)** **MRI spinal no (*N* = 28)**	49.53 ± 14.29	0.56
	48.21 ± 17.13	
	**SDMT Z-Scores** **Mean ±SD**	***p*-Value**
**Gd+ lesion (*N* = 40)** **Gd− lesion (*N* = 41)**	−0.25 ± 1.24 −0.6 ± 1.34	0.22
**High brain LL yes (*N* = 46)** **High brain LL no (*N* = 23)**	−0.65 ± 1.4 −0.13 ± 1.07	***0.049***
**MRI spinal yes (*N* = 53)** **MRI spinal no (*N* = 28)**	−0.38 ± 1.22	0.59
	−0.50 ± 1.44	

Abbreviation: Gd+, presence of gadolinium-enhanced lesions; Gd−, absence of gadolinium-enhanced lesions; IQR, interquartile range; LL, lesion load; MRI, magnetic resonance imaging; SD, standard deviation; SDMT, Symbol Digit Modalities Test.

## Data Availability

Datasets generated are available from the corresponding author on reasonable request.

## Supplementary Materials

The following are available online at https://www.mdpi.com/article/10.3390/brainsci11111521/s1: Table S1. Multiple regression analyses.
