# Supplementary material for: Serum Vitamin D as a Marker of Impaired Information Processing Speed and Early Disability in Multiple Sclerosis Patients
Source: Brain Sci. 2021 Nov 17;11(11):1521. doi: 10.3390/brainsci11111521 (PMC8615879; doi:10.3390/brainsci11111521)
Supplement: Supplementary file 1 [file brainsci-11-01521-s001.zip › brainsci-1442473-SI.pdf]

SUPPLEMENTARY MATERIAL

Table S1. Multiple regression analyses.

Multiple regression analyses performed to identify the best predictors of slowed IPS. The model includes vitamin D level, age at diagnosis, gender, educational level, EDSS at diagnosis, type of MS, and MRI characteristics (brain, spinal cord, and gadolinium) at baseline as independent variables; SDMT raw value as dependent variables. *Abbreviations:* df : degrees of freedom, EDSS: expanded disability status score, gd : gadolinium, MS : multiple sclerosis, SC spinal cord , SDMT: symbol digit modalities test, SE: standard error, WMLL white matter lesion load.

Model Summary

				Change statistics				
R	R2	adjusted R2	SE of the estimate	R2 change	F change	df1	df2	Sign. F change
,617[a]	,381	,302	12,73025	,381	4,846	9	71	,000

a. Predictors: (costant), EDSS at diagnosis, brain WMLL, Gd status, SC involvement, gender, age at diagnosis, educational level, MS subtype, Vitamin D

ANOVA[a]

	Sum of Squares	df	Mean square	F	Sign.
Regression	7067,354	9	785,262	4,846	,000[b]
Residual	11506,201	71	162,059		
Total	18573,556	80			

a. Dipendent variable: RAW SDMT

b. Predictors: (costant),EDSS at diagnosis, brain WMLL, Gd status, SC involvement, gender, age at diagnosis, educational level, MS subtype, Vitamin D

Coefficients[a]

	Unstandardized coefficients		Standardized coefficients			95% CI for B	
	B	St. Error	Beta	t	Sign.	Lower bound	Upper bound
(Costant)	25,423	16,603		1,531	,130	-7,682	58,529
Vitamin D	,465	,178	,267	2,607	,011	,109	,820
brain MRI WMLL	-4,275	3,065	-,140	-1,395	,168	-10,387	1,837
Gd status	2,474	2,986	,082	,828	,410	-3,480	8,427
SC involvement	,122	3,115	,004	,039	,969	-6,090	6,334
Educational level	1,616	,514	,346	3,148	,002	,593	2,640
Gender	1,755	3,337	,055	,526	,601	-4,900	8,409
MS subtype	1,472	2,355	,064	,625	,534	-3,224	6,167
Age at diagnosis	-,315	,130	-,244	-2,423	,018	-,574	-,056
EDSS at diagnosis	2,588	2,110	,146	1,226	,224	-1,620	6,796

a. Dipendent Variable: RAW SDMT

Multiple regression analyses performed to identify the best predictors of slowed IPS (when condidering corrected values for age, gender and educational level). The model includes vitamin D level, EDSS at diagnosis, type of MS, and MRI characteristics (brain, spinal cord, and gadolinium) at baseline as independent variables; SDMT Z-score as dependent variable. *Abbreviations:* df : degrees of freedom, EDSS: expanded disability status score, gd : gadolinium, MS : multiple sclerosis, SC spinal cord , SDMT: symbol digit modalities test, SE: standard error, WMLL white matter lesion load.

Model Summary

R	R2	adjusted R2	SE of the estimate	Change statistics				
				R2 change	F change	gl1	gl2	Sign. F change
,425[a]	,180	,114	1,22388	,180	2,715	6	74	,019

a. Predictors: (costant), EDSS at diagnosis, brain MRI WMLL, Gd status, SC involvement, MS subtype, vitamin D

ANOVA[a]

	Sum of squares	gl	Mean square	F	Sign.
Regression	24,400	6	4,067	2,715	,019[b]
Residual	110,843	74	1,498		
Total	135,242	80			

a. Dipendent Variable: SDMT Z-score

b. Predictors: (costant), EDSS at diagnosis, brain MRI WMLL, Gd status, SC involvment, MS subtype, vitamin D

Coefficients[a]

	Unstandardized coefficients		Standardized coefficients			95% CI for B	
	B	SE	Beta	t	Sign.	Lower bound	Upper bound
(Costant)	-1,246	,867		-1,438	,155	-2,973	,481
Vitamin D	,052	,017	,351	3,122	,003	,019	,085
brain MRI WMLL	-,506	,288	-,194	-1,760	,083	-1,079	,067
Gd status	,353	,279	,136	1,265	,210	-,203	,908
SC involvement	,037	,294	,014	,126	,900	-,549	,622
MS subtype	-,108	,216	-,055	-,502	,617	-,538	,321
EDSS at diagnosis	,088	,181	,058	,484	,630	-,273	,449

a. Dipendent Variable: SDMT Z-score
